# The Potential Properties of Natural Compounds in Cardiac Stem Cell Activation: Their Role in Myocardial Regeneration

**DOI:** 10.3390/nu13010275

**Published:** 2021-01-19

**Authors:** Cristina Carresi, Miriam Scicchitano, Federica Scarano, Roberta Macrì, Francesca Bosco, Saverio Nucera, Stefano Ruga, Maria Caterina Zito, Rocco Mollace, Lorenza Guarnieri, Anna Rita Coppoletta, Micaela Gliozzi, Vincenzo Musolino, Jessica Maiuolo, Ernesto Palma, Vincenzo Mollace

**Affiliations:** 1 Institute of Research for Food Safety & Health IRC-FSH, University Magna Graecia, 88100 Catanzaro, Italy; federicascar87@gmail.com (F.S.); robertamacri85@gmail.com (R.M.); boscofrancesca.bf@libero.it (F.B.); saverio.nucera@hotmail.it (S.N.); rugast1@gmail.com (S.R.); mariacaterina.zito@libero.it (M.C.Z.); rocco.mollace@gmail.com (R.M.); lorenzacz808@gmail.com (L.G.); annarita.coppoletta@libero.it (A.R.C.); micaela.gliozzi@gmail.com (M.G.); v.musolino@unicz.it (V.M.); jessicamaiuolo@virgilio.it (J.M.); palma@unicz.it (E.P.); mollace@libero.it (V.M.); 2 Nutramed S.c.a.r.l., Complesso Ninì Barbieri, Roccelletta di Borgia, 88100 Catanzaro, Italy

**Keywords:** cardiac stem cells, heart failure, natural compounds, myocardial regeneration

## Abstract

Cardiovascular diseases (CVDs), which include congenital heart disease, rhythm disorders, subclinical atherosclerosis, coronary heart disease, and many other cardiac disorders, cause about 30% of deaths globally; representing one of the main health problems worldwide. Among CVDs, ischemic heart diseases (IHDs) are one of the major causes of morbidity and mortality in the world. The onset of IHDs is essentially due to an unbalance between the metabolic demands of the myocardium and its supply of oxygen and nutrients, coupled with a low regenerative capacity of the heart, which leads to great cardiomyocyte (CM) loss; promoting heart failure (HF) and myocardial infarction (MI). To date, the first strategy recommended to avoid IHDs is prevention in order to reduce the underlying risk factors. In the management of IHDs, traditional therapeutic options are widely used to improve symptoms, attenuate adverse cardiac remodeling, and reduce early mortality rate. However, there are no available treatments that aim to improve cardiac performance by replacing the irreversible damaged cardiomyocytes (CMs). Currently, heart transplantation is the only treatment being carried out for irreversibly damaged CMs. Hence, the discovery of new therapeutic options seems to be necessary. Interestingly, recent experimental evidence suggests that regenerative stem cell medicine could be a useful therapeutic approach to counteract cardiac damage and promote tissue regeneration. To this end, researchers are tasked with answering one main question: how can myocardial regeneration be stimulated? In this regard, natural compounds from plant extracts seem to play a particularly promising role. The present review will summarize the recent advances in our knowledge of stem cell therapy in the management of CVDs; focusing on the main properties and potential mechanisms of natural compounds in stimulating and activating stem cells for myocardial regeneration.

## 1. Introduction

Cardiovascular diseases (CVDs) are the leading cause of death in the developed world; accounting for approximately 30% of deaths globally, according to the World Health Organization, [1], thus, representing one of the main health problems worldwide [2,3]. The increase in life expectancy, together with several risk factors such as smoking, poor nutritional habits, sedentary lifestyle, obesity, high blood pressure, and diabetes is closely linked to the onset of CVDs [2,3,4]. To date, the number of people with CVDs in most European countries and various other countries around the world, moving a volume of billions of dollars in healthcare [5,6,7,8], is expected to further increase according to the American Heart Association reports [7].

Among CVDs, ischemic heart disease (IHD), such as myocardial infarction (MI), is one of the major causes of morbidity and mortality throughout Europe, America and Asia [5,9,10]. When myocardial ischemia occurs, the main consequence is represented by irreversible cell death, which is accompanied by the loss of viable myocardial tissue, pathological remodeling and scar tissue formation; dramatically predisposing to heart failure (HF) [11]. In this condition, “time” represents the main discriminant factor on which current pharmacological and surgical therapies focus, in order to reduce serious adverse events and ultimately the mortality rate.

Although traditional therapeutic options are highly efficient in improving symptoms, decelerating adverse cardiac remodeling and reducing early mortality rate, there is no available treatment that aims to improve cardiac performance by replacing the irreversible damaged CMs [12].

It is now clear that despite the adult heart being composed of mainly terminally differentiated cells, unable to re-enter the cell cycle, it cannot be considered a terminally differentiated organ because it also contains stem cells that support its regeneration [13]. However, the loss of CMs due to ischemic heart events is currently counteracted only by heart transplantation [11].

Over the last two decades, new alternative therapies have been studied to address the underlying problem of CM loss and improve the clinical condition of patients. Among these, stem cell therapy has proven to be a promising therapeutic approach for the treatment of IHDs [14]. Stem cells possess specific features of self-renewing, clonogenicity, and differentiation into specialized cell types under appropriate conditions [15]. Stem cells are commonly divided into various major subgroups such as embryonic stem cells (ESCs), induced pluripotent stem cells (iPSCs), and adult or somatic stem cells. The latter include bone marrow stem cells (BM-SCs), (which in turn consist of hematopoietic stem cells -HSCs, bone marrow derived mesenchymal stem cells -BM-MSCs, multipotent adult progenitor cells -MAPCs and endothelial progenitor cells -EPCs), mesenchymal stem cells (MSCs), cardiac stem cells (CSCs), and skeletal myoblasts [15]. During the last twenty years, several pre-clinical studies have revealed the great potential and also the serious limitations in the use of different type of stem cells, such as ESCs [16,17,18], iPSCs [19,20,21], or adult/somatic stem cells [22,23] in the treatment of CVDs. Moreover, many advances have been made to enable the application of stem cell therapy in patients with CVDs through the implementation of different clinical trials [22,24,25,26,27,28].

In short, today knowledge on the use of stem cells in regenerative therapy is manifold. Several animal studies and early clinical trials clearly show the feasibility of the stem cell-based therapies and the beneficial effects of numerous stem cell types in the regeneration of injured myocardium. However, the beneficial effects of stem cell-based treatment on myocardial performance are still quite limited by the low rates of engraftment and cell survival. These limitations are due to the microenvironment of the damaged site of cardiac tissue which is often associated with inflammatory response, oxidative stress, and extracellular matrix (ECM) degradation [11]. Therefore, it is essential to solve the main issue of understanding how to boost the efficacy of stem cell repair to make cardiac regenerative therapy an effective therapeutic tool.

To date, many different methods to improve the efficiency of stem cell therapy and to better understand their way of action have been investigated. Among these, there are several cell-delivery methods which include intra-myocardial, intra-coronary or intra-venous injection of stem cells. However, using these methods, the survival rate of injected cells was below 1% [29,30,31]. Both cell-sheet technology and the injectable hydrogels offer more advantageous cell-delivery systems by reducing the mechanical damage to the host myocardium; thus improving the survival rate of the transplanted cells [32,33]. In addition, continuous advances in genetically engineered stem cell techniques, such us the overexpression of cytoprotective or anti-apoptotic genes, are enhancing the outcomes of the stem cell therapy [34,35]. Before transplantation, cell preconditioning strategies such as culturing the cells with agonists, exposing cells to hypoxia, heat shock, or to oxidative stress have also been used [36,37,38]. The mechanisms of cardiac repair by transplanted stem cells represent another important issue which is not yet fully understood [11]. The scientific discussion about the possible mechanisms of action underlying the ability of stem cells to regenerate damaged heart tissue is highly debated. Suggested mechanisms include the direct cardiomyogenic differentiation of the transplanted stem cells and progenitor cells, after isolation from autologous or allogeneic source tissues, and the paracrine effect of stem cells, which indirectly stimulate the regenerative process through secretion of soluble cytokines and growth factors [39]. Interestingly, the indirect paracrine effect of transplanted stem cells also seems to stimulate the activation and differentiation of resident CSCs [40].

Nowadays, the paracrine effect on stem cells has been identified as a key factor of stem cell-therapy efficacy. Indeed, it has been shown that paracrine signaling enables transplanted stem cells to protect the surrounding cardiovascular tissue due to the release of different growth factors, such as transforming growth factor (TGF)-β, vascular endothelial growth factor (VEGF), fibroblast growth factor (β-FGF), hepatocyte growth factor (HGF), insulin-like growth factor 1 (IGF-1), stromal cell-derived factor (SDF)-1, epidermal growth factor (EGF), thymosin b4, and a variety of cytokines [41].

It is now well-established that the secretion of biologically active molecules from transplanted stem cells leads to the activation of angiogenic, antiapoptotic and antifibrotic paracrine patterning within the damaged heart; these actions, also referred to as “trophic effects”, play a major role in tissue repair [42]. Moreover, the administration of stem cell-conditioned medium has shown beneficial effects, comparable to those of the transplanted cells. These beneficial effects include promoting regenerative processes, protecting the existing CMs from damage and death, reducing matrix remodeling and enhancing the recruitment of circulating progenitor cells, and activation of cell proliferation and CM differentiation [43,44]. A major role in the paracrine effect of stem cells seems to be played by a type of extracellular vesicles called exosomes. Stem cell-secreted exosomes, function as cargo of nucleic acids, proteins, lipids, and several cardio-protective and pro-angiogenic mRNAs and non-coding RNAs; suggesting their critical role in exchange of biological information and clinical usefulness as biomarkers of CVDs and as therapeutic tools [45,46]. Indeed, it was recently shown that direct injection of exosomes derived from stem cells into a damaged heart decreases the systemic inflammation, oxidative stress, and significantly improves the regenerative effects produced by stem cells [47].

Interestingly, pharmacological preconditioning represents a novel and efficient technique for stimulating the secretory activity of stem cells. For example, in vitro and in vivo studies reported the effectiveness of oxytocin in stimulating the release of cytokines [48] and the anti-ischemic effect of trimetazidine and sildenafil, when applied to stem cells prior to transplantation [49,50]. Moreover, drug-mediated activation or inhibition of pathways can modify stem cell physiology; enhancing cell survival and engraftment thereby improving cardiac regeneration [51].

**Figure 1:**
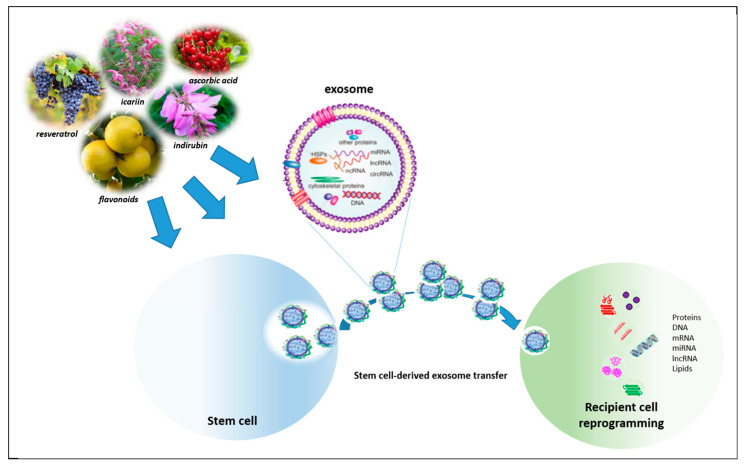
Potential properties of natural compounds in the stimulation of stem cell paracrine signaling. Some natural compounds (ascorbic acid, indirubin, resveratrol, icariin, bergamot polyphenols) emerging as useful tools in improving homing, engraftment, self-renewal and stem cell cardiovascular lineage specification, possibly, through paracrine signaling regulation. Stem cells exert paracrine effects on neighboring somatic cells via the secretion of micro-vesicles, such as exosomes, containing hundreds of different proteins, mRNAs and miRNAs under normal conditions or in response to cellular stress. Transferring the content of stem cell-derived exosomes can facilitate reprogramming of the recipient somatic cell.

In this regard, the use of natural compounds from plant extracts seems to play a particularly intriguing role. Several studies have provided a number of molecules able to selectively control stem cell growth and differentiation. Natural compounds such as: ascorbic acid, indirubin, resveratrol, icariin, bergamot polyphenols, are emerging as useful tools capable of orchestrating the homing, engraftment, and survival of cells; improving self-renewal and modulating stem cell cardiovascular lineage specification, possibly through regulation of paracrine signaling (Figure 1).

The present review briefly summarizes the knowledge on stem cell therapy in the management of CVDs and focuses on the potential properties of natural compounds in stimulating and activating stem cells for myocardial regeneration.

## 2. Cardiac Regeneration and Stem Cells Therapy

The presence of damaged and necrotic heart tissue, with stromal connective tissue formation and deposition of fibrotic scar are the main features of IHDs and particularly of MI. These conditions, as well as in non-IHDs, are characterized by a loss of CMs, expansion of the ECM and increase in hypertrophic CMs as an attempt to compensate for the reduced cardiac function. Persistence of pathological stress, cardiac remodeling and development of HF with poor prognosis are treated with limited therapeutic intervention such as ventricular assist devices and cardiac transplantation [52,53,54]. In addition, targeted pharmacological treatments are used to counteract HF development [52,53,54]. In this regard, research on new strategies to prevent cardiac damage and dysfunction has focused on cardiac regeneration by promoting cell-based therapy, mainly based on transplantation, into the damaged heart, of several stem cell types or in vitro stem cell derived- CMs, endorsing the regeneration of resident CSCs [54]. Originally, the heart was considered a terminally differentiated organ, unable to replicate and to be responsive to myocardial detriment [55,56,57,58]. After the discovery of the regenerating adult mammalian heart’s capacity the potential replicative activity of CMs was investigated [55,56,57,58]. This ability was observed in normal and pathological condition, by the presence of cycling ventricular myocytes that rise in case of damage, in contrast with the idea that after birth CMs lost the possibility to divide and regenerate, giving rise to the question about the origin of these cycling myocytes [58,59]. The studies about putative CSCs, showed that they shared certain features with stem cells such as the markers on the cell surface like the stem cell factor receptor (c-kit), the Stem cells antigen-1 (Sca1), the multidrug resistance gene product-1 (MDR-1), the capacity to self-renewing, generate clones and differentiate into myocytes, smooth muscle cells and endothelial cells [58,60]. However, CMs turnover is only 0.5% to 2% per year and was found to be insufficient to supply the necessity of a damaged heart, leading to terminal HF without a proper intervention [61]. Therefore, new alternative therapies were investigated to address the underlying problem of CM loss and improve patients’ clinical condition. Among all, stem cell therapy has proven to be a promising therapeutic approach for the treatment of IHDs [14]. The intriguing capability of stem cells to support cardiac repair has been well investigated in many in-depth studies [62,63,64].

Several preclinical studies aimed to investigate the characteristics of different stem cell types in cardiac damage, including ESCs, iPSCs and adult or somatic stem cells, showing the possibilities of improving cardiac dysfunction and promoting tissue regeneration [65,66,67], translating this knowledge in early clinical trials [68,69]. Despite this evidence and the observation that after cardiac injury it is possible to observe cardiac recovery [70], the data about what type of cells robustly contributes to cardiac regeneration, and which provides the real therapeutic benefit eventually deriving from the use of stem cells, is still under debate [71]. The main results obtained using different stem cell types in in vitro and in vivo studies and in the early clinical trials are summarized below and in Table 1.

### 2.1. Embryonic Stem Cells

Embryonic stem cells (ESCs) are pluripotent cells derived from the inner cell mass of pre-implantation blastocysts of a murine or human host. ESCs showed the ability to discriminate in cells of all three germ layers, thus, including the generation of cardiac myocytes [40,72,73]. From early studies conducted on different animal models of acute MI and IHDs, such as pig or guinea pig, the injected ESCs have shown the ability to act as a biological pacemaker in electro-physiologically silenced or atrioventricular blocked hearts [74,75]. However, in these experiments, ESCs give rise not only to CMs, but also to other non-cardiac cell types. For this reason, the need arose to achieve a more specific ESC differentiation into CMs [74,76]. Over the last decades, numerous procedures have clearly demonstrated that ESCs, adequately stimulated, express markers, such as Receptor Tyrosine Kinase-Like Orphan Receptor2 (ROR2), CD13, Vascular Endothelial Growth Factor Receptor 2 (VEGFR-2), Platelet-Derived Growth Factor Receptor-α (PDGFR-α) and can differentiate into endothelial cells, vascular smooth muscle cells and CMs in vitro [77,78]. It was observed that the use of different growth factors or cytokines, such as activin, bone morphogenetic proteins (BMPs), β-FGF, TGF-β, or tumor necrosis factor-α (TNF-α) could be employed to efficiently generate CMs from ESCs [75,79]. Moreover, manipulating the specific cardiac signal pathway, such as with the use of a glycogen synthase kinase 3 (GSK3) inhibitor followed by a chemical inhibitor of wnt signaling, makes the differentiation more specific [76,80]. Subsequent several preclinical investigations have been well demonstrated that ESCs when transferred into the infarcted myocardium well-differentiate, produce CM grafts and leads to a significant improvement in left ventricular function [16,17]. The injection of ESC-derived CMs into rat myocardium was able to reduce the severity of HF after MI and ameliorate left ventricular fractional shortening (FS), ejection fraction (EF) and left ventricular regional wall motion, improving cardiac function [81]. Furthermore, a limited number of CMs derived from ESCs were observed to survive after transplantation in animal trials, forming desmosome and gap-junction after several weeks [71]. A further study conducted on non-human primates with MI showed remuscularization of the infarcted heart with the formation of electromechanical junctions and ventricular myocytes able to restore cardiac function [82]. Nevertheless, despite the documented capability of ESCs in cardiac regeneration their clinical application as a treatment modality in patients clashes with some important ethical issues and biological considerations [25]. For this reason, many countries have imposed prohibitions on research funding and on the use of human ESCs (hESCs) derived from an embryo destruction [18]. In addition, some studies have identified the ability of ESCs to generate teratomas (tumors of mixed germ layers) and graft refusal when transplanted undifferentiated [73], together with genetic variability and the risk of immune rejection due to their nature of pluripotency and allogeneic cell type [83]. Many efforts to enhance purifications and differentiations protocols are ongoing [84]. Recently, hESC-derived differentiated cells expressing cardiac transcription factors Insulin gene enhancer protein (Isl-1) and Stage Specific Embryonic Antigen-1 (SSEA-1) when transplanted into the infarct site of 68-year-old patients with impaired left ventricular EF showed significant improvement in left ventricular function without complications like arrhythmias, tumor creation, or detrimental immunosuppression [24]. The feasibility and safety of hESCs were recently evaluated in an early trial of six patients with severe ischemic left ventricular dysfunction. Human ESC-derived cardiovascular progenitors embedded in a fibrin patch were epicardially delivered during a coronary artery bypass procedure leading to an increased systolic motion of the cell-treated segments without the identification of tumor formation or signs of arrhythmias during follow-up [85]. However, ESCs remain more difficult to manage than the more recently discovered iPSCs, which have been suggested as potential substitutes for hESCs.

### 2.2. Induced Pluripotent Stem Cells

Induced PSCs are produced from adult somatic cells over a genetic reprogramming process [86], capable of obtaining cells with properties similar to those of hESCs, reaching a solution ethically shared by the scientific community [87]. The ease of sampling and propagation of iPSCs has made it possible to draft advanced reprogramming protocols [88] and established models of CVDs [89]. After the discovery of iPSCs in 2006 [90], several studies have successfully differentiated them into many distinct cell types, yielding in vitro model specific for tissue, disease, and type of patient [91]. Due to the overexpression of several transcription factors, such as Kruppel-like factor 4 (Klf4), SRY-box transcription factor 2 (Sox2), cellular myelocytomatosis oncogene (c-Myc), Octamer-binding transcription factor 4 (Oct3/4), iPSCs become similar to ESCs and able to indefinitely proliferate in vitro, form beating embryoid bodies (EBs) and differentiate into all somatic cells including smooth muscle cells, endothelial cells and CMs. In particular, CM derived from iPSCs (iPSC-CMs) express cardiac specific markers GATA-binding protein (GATA-4), GATA-2, NK2 transcription factor related locus 5 (Nkx2.5), showing mature electrophysiological function [92,93]. During the last decade, methods for differentiating iPSCs into beating CMs (iPSC-CMs) have been efficiently optimized [94]. Evidence from preclinical studies has shown the capacity of iPSCs-CMs to restore cardiac contractility. Transplantation of iPSCs-CMs into non-human primate model of MI leads to partial remuscularization of the scar tissue promoted by grafted CMs [95]. Moreover, in a mouse model of acute MI, the injection of iPSCs ameliorates left ventricular function and attenuates cardiac remodeling [96]. Importantly, CMs derived from human iPSCs (hiPSC-CMs) have been shown to replicate the genome of the patient donor [97], contract [98] and successfully engraft into a host organism [19]. Although, the resulting cells remain qualitatively and quantitatively immature in their structure and function, particularly showing fetal gene expression, disorganized morphology, immature calcium processing and ultrastructural and electrophysiological features [99,100]. Recently, many approaches have been successfully developed to induce a more defined maturation of hiPSC-CMs leading to substantial progress in addressing this limitation [101]. These include the use of small molecules combined with hormones [102] electrical and/or mechanical stimulation [103] together with the use of conductive materials [104] and the development of three-dimensional (3D) culture conditions or organoids [105]. Despite the previously discussed gaps in structural and electrophysiological characters of hiPSC-derived and adult CMs, several studies have proven the exceptional perspective that the use of hiPSC offers for cardiovascular disease modelling and in treatment approaches based on personalized medicine [25]. Furthermore, innovative methods, such as transitory expression of the reprogramming factors [106], have been defined to avoid the onset of teratoma, due to the pluripotent feature of iPSCs [20,21].

### 2.3. Bone Marrow Derived Cells

Nowadays, the existence of stem cell niches, residing in different organs and tissues of the body, is well established. These cells, defined as adult stem cells, are multipotent stem cells (e.g., BMSCs, CSCs), which can differentiate into limited lineages, with a greater regenerative potential in organs/tissues with a high cell turnover and in response to injury. Adult stem cells have been extensively studied in the last 20 years proving to be safe and effective in myocardial regeneration process thanks to their high degree of plasticity [15]. Bone marrow derived stem cells (BMSCs) are multipotent stem cells derived and purified from patients through bone marrow aspiration [107]. These cells are made up of heterogeneous population of different cell types, such as hematopoietic stem cells, endothelial stem/progenitor cells and mesenchymal stem cells [108]. As early as 2001, one of the first pre-clinical studies reported the regenerative effects of BMSCs negatively selected for blood lineage markers (Lin^−^) and positively selected for c-kit (c-kit^+^) in the repair of infarcted cardiac tissue and in the improvement of ventricular function [109]. Indeed, Lin^−^c-kit^+^ BMSCs locally transplanted into the peri-infarcted left ventricle of a murine model of coronary ligation differentiate into cardiac tissue reducing the infarcted area thanks to the formation of new myocytes, endothelial cells and smooth muscle cells generating de novo myocardium, inclusive of coronary arteries, arterioles and capillaries [109]. Afterwards, other important studies demonstrated the ability of BMSCs to differentiate into CMs and vascular cells [110], leading to a rapid translation of stem cell therapies in the clinical setting. Indeed, in 2002 the first autologous intracoronary mononuclear BMSC transplantation was performed in patients with MI showing a clear therapeutic effect related to myocardial regeneration and neovascularization due to BMSCs [22]. BMSC transplantation was also performed in patients with IHDs improving cardiac remodeling and function and reducing infarct size and the resulting incidence of death [111,112]. However, the benefits obtained in the clinical practice are controversial. Indeed, some other trials have shown that the administration of BMSCs did not improve long term left ventricular function and remodeling in patients with MI or with non-ischemic dilated cardiomyopathy [113,114]. It was also reported that there is no clear in vivo evidence on the ability of BMSCs to differentiate into CMs [115]. Furthermore, the mechanism underlying the benefits observed in treated patients is not well-understood and it has been proposed that the latter cannot be due to a direct effect of the limited number of injected BMCs, but instead relates to the activity of the released growth factors by BMSCs [108].

### 2.4. Mesenchimal Stem Cells

Mesenchymal stem cells (MSCs) are stem cells capable of forming colonies, in vitro, and of differentiating into different cell types, such as osteocytes, chondrocytes, adipocytes, myocytes, and other lineages [116]. MSCs derive from different sources, and more commonly, research has focused on those of the bone marrow, adipose tissue origin [117]. MSCs have shown beneficial effects in different MI model of pre-clinical and clinical studies, improving systolic and diastolic function through both paracrine effects and trans-differentiation into CMs, vascular endothelium and smooth muscle cells [118,119,120,121,122]. Although the mechanism by which MSCs restore the myocardium has not been established, it has been proposed that MSC homing into damage heart invading the ECM [123], while the paracrine and immunomodulatory effects are the main mechanisms proposed. These two mechanisms are related to the secretion of various cytokines like VEGF, β-FGF, interleukin (IL)-6, HGF, SDF-1, and IGF-1 and to the ability of MSCs to mobilize autologous CSCs or progenitor cells to differentiate into CMs and proliferate [124,125]. However, experiments with the use of MSCs have encountered several side effects: The delivery of MSCs often leads to the formation of calcified or ossified structures after injection and cardiac sarcoma or other tumors formation has been observed after transplantation of unmodified MSCs in the peri-infarct area of the heart, suggesting that more eligible methods of delivery, differentiation and cell survival are needed to promote survival of transplanted MSCs [125].

### 2.5. Cardiac Stem Cells

More recently, the endogenous regenerative capacity of the heart has been an area of extensive research. The discovery that the adult heart contains a pool of cardiac stem and progenitor cells able to regenerate CM population and coronary vessels has been extensively studied [126,127,128]. Initially, primitive cells that express c-kitSca-1 and MDR-1 were identified in mammalian heart, and importantly, in human control hearts [129,130]. In 2003, for the first time, Beltrami and colleagues identified a pool of endogenous cardiac stem cell in the adult mammalian myocardium as a small cell population that does not express blood lineage markers (Lin^−^), and likewise stem cells, express c-kit [59,60]. This specific cardiac stem cell population, mainly displayed as niches and localized in the atria and ventricular apex, has been shown to be self-renewing, clonogenic and multipotent [59,60]. Moreover, fate mapping studies well-demonstrated that resident cardiac stem and progenitor cells contribute to adult cardiomyogenesis by direct differentiation [13,23]. Several preclinical studies showed that CSCs or cardiac progenitor cells (CPCs) when inoculated into the infarcted myocardium are able to replenish the damaged heart tissue (scar size) due to their proliferative capacity, re-establishing cardiac structure and activity and improving left ventricular function [131]. The regenerative capacity seems to be connected with mechanisms of excretion of cytokines and growth factors by the exogenous CSCs that would lead to the activation of endogenous CSC pool, their increased proliferation and commitment into adult cardiac cells [68,132]. It was detected that treatment with IGF-1 and HGF, in a large animal model of acute myocardial infarction (AMI), was able to activate CSCs promoting cardiac muscle regeneration, ameliorating cardiac function and reducing fibrosis and hypertrophic reaction, probably thanks to the resulting paracrine effects [133]. More recently, an in depth study demonstrated well that clones derived from Lin^−^ c-kit^+^ CSCs showed a full transcriptome and protein expression, as well as the presence of sarcomere structures, spontaneous contraction and electrophysiological properties of differentiated CMs, after an adequate stimulation with TGF-β/wnt molecules [134]. In addition, this specific pool of CSCs, when injected into a murine infarcted myocardium, can replenish damaged CMs and contribute to the development of capillaries, possibly through activation of the SMAD2 pathway, which is involved in the commitment of CM lineage [134].

The experimental evidence obtained in different animal models has stimulated several clinical trials in patients with MI or IHDs. In 2011, phase one of the first clinical trial performed on patients with ischemic cardiomyopathy began [26]. Interesting data emerged from the aforementioned and other clinical studies that followed, highlighting the beneficial effects of CSCs delivery in reducing infarct size, improving global heart function, contractility, systolic wall thickening and cardiac fibrosis [27,135,136,137]. Some studies have also shown that intracoronary injection of autologous CSCs [27] and also of allogeneic CSCs was safe and feasible [28]. However, the applicability of autologous CSCs in human cell therapy would be limited mainly due to patient-specific invasive myocardial biopsy and cell processing time and the resulting delay in therapy administration. Conversely, the use of allogeneic CSCs avoids logistical and economic constraints [28]. Nevertheless, the actual significance of CSCs in cardiac cell repair, particularly in adults after injury, is still widely debated [138]. Some contradictory reports have been reported on a specific subgroup of endogenous CSCs, mainly identified by the expression of c-kit, discussing the extent of CSC regenerative capacity and even the reliability of the methods used to quantify it [139,140].

In conclusion, to date, there is no unanimous opinion about the intrinsic endogenous regenerative capacity of the adult mammalian heart and subsequent studies will be necessary to better understand the underlying mechanism of the regenerative response and to develop clear protocols of myocardial regeneration and protection of cardiac function [141].

**Table 1:** Stem cell properties in cardiac regeneration.

Stem Cell Type	Properties	In Vitro/In Vivo Models	Clinical Trials	References
Embryonic stem cells (ESCs)	Differentiate into CMs Engraftment of ESC-derived CMs: ○ ↑ LV functions after MI ESCs-derived CMs: ○ ↓ HF post MI ○ ↑ LVFS, LVEF, LV ○ wall motion ESCs-derived CMs determine: ○ deformation of desmosome and gap-junction after MI ESCs-derived CMs: ○ ↑ remuscolarization of infarcted heart ○ ↑ formation of electromechanical junction ○ ↑ formation of ventricular myocytes ○ ↑ cardiac functions ↑ hESCs-derived CPCs: ○ ↑ systolic motion of the treated segments	ESCs treated with: BMP4, β-FGF, TGFβ, TNF-α, wnt inhibitors or GSK3 inhibitors MI in mice and rats MI in guinea pig or pig MI in non-human primates	infarct site of a 68-year-old patients with impaired LVEF patients with severe ischemic LV dysfunction.	[24,71,74,75,76,79,82,85]
Induced Pluripotent stem cells (iPSCs)	indefinitely proliferate form beating embryoid bodies differentiate into all somatic cells, including CMs iPSC-CMs express cardiac specific markers GATA-4, GATA-2, Nkx2.5, showing mature electrophysiological function iPSCs-CMs: ○ ↑cardiac contractility, ○ ↑ remuscularisation of the scar tissue ○ ↑ cadherins and gap junctions ○ ↑ FS injection of iPSCs: ○ ↑ LV function ○ ↓ cardiac remodelling hiPSC-CMs: ○ replicate the genome of the patient donor contract successfully engraft in a host organism	iPSCs treated with Klf4, Sox2, c-Myc, Oct3/4 I in non-human primate acute MI in mice LAD-ligated mice 3D Cultured iPSCs	iPSC-derived CMs transplanted in patients after MI	[19,74,92,93,95,96,97]
Bone marrow derived cells (BMSCs)	↑ ventricular function ↑ myocardial regeneration ↑ neovascularization ↓ cardiac remodelling ↓ infarct size ↓ incidence of death	coronary ligation in mice MI in mice	MI in patients IHDs in patients	[22,109,110,111,112]
Mesenchimal stem cells (MSCs)	↑cardiac function ↑ systolic and diastolic function through paracrine effect and trans- differentiation into CMs, VECs and SMCs. MSCs homing into damage heart: ○ invading ECM ○ secrete various cytokines (VEGF, IL-6, β-FGF, HGF, SDF-1, IGF-1) ○ mobilize autologous CSCs or progenitor cells to differentiate into CMs	cultured CMs from neonatal rats exposed to H/R and treated with MSC medium MI in pigs	MSCs ○ intracoronary injection in patients with systolic heart failure	[118,119,120,121,122,123,124,125]
Cardiac stem cells (CSCs)	proliferate and differentiate into cardiomyogenic lineage under specific differentiation conditions ↑ cardiac muscle regeneration, ↑ cardiac function ↓ fibrosis ↓ hypertrophic reaction CSCs or CPCs when inoculated into the infarcted myocardium: ○ ↓ scar size, ○ ↑ cardiac structure and activity ○ ↑ LV function exogenous CD45^−^c-kit^+^ CSCs excreted cytokines and growth factors: ○ ↑ activation of endogenous CD45^−^ ○ c-kit^+^ CSCs ↑ proliferation ○ ↑ commitment into adult cardiac cells c-kit^+^ CSCs delivery: ○ ↓ infarct size ○ ↑ global heart function ○ ↑ contractility ○ ↓ systolic wall thickening ○ ↓ cardiac fibrosis specific Lin^−^c-kit^+^ cloned progeny injected into the infarcted myocardium: ○ ↑ CSC engraftment rate ○ ↑ formation of new CMs, arterioles, ○ capillaries ○ ↓ myocyte apoptosis, hypertrophy ○ ↓ scar size, LV dilation ○ ↑ FS, EF	CD45^−^c-kit^+^ CSCs treated with IGF-1 and HGF AMI in large animals MI in murine models	patients with ischemic cardiomyopathy (SCIPIO trial) patients with ventricular dysfunction after MI (CADUCEUS trial) CPCs infusion in patients with univentricular heart disease (PERSEUS trial)	[13,23,26,27,58,59,60,137]

↑ increase, ↓ decrease. CMs: cardiomyocytes; LV: left ventricle; MI: myocardial infarction; HF: heart failure; LVFS: left ventricular fractional shortening; LVEF: left ventricular ejection fraction; hESC: human embryonic stem cell; CPC: cardiac progenitor cell; BMP4: bone morphogenetic protein-4; β-FGF: fibroblast growth factor-β, TGF-β: transforming growth factor-β; TNF-α: tumor necrosis factor-α; GSK3: glycogen synthase kinase 3; iPSC-CMs: induced pluripotent stem cell-derived cardiomyocytes; GATA-4: GATA-binding protein 4; GATA-2: GATA-binding protein 2; Nkx2.5: NK2 transcription factor related locus 5; hiPSC-CMs: human induced pluripotent stem cell-derived cardiomyocytes; Klf4: Kruppel-like factor 4; Sox2: SRY-box transcription factor 2; c-Myc: cellular myelocytomatosis oncogene; Oct3/4: Octamer-binding transcription factor 3/4; LAD: left coronary artery ligation; IHDs: ischemic heart diseases; VECs: vascular endothelial cells; SMCs: smooth muscle cells; ECM: extracellular matrix; VEGF: vascular endothelial growth factor; IL-6: interleukin-6; HGF: hepatocyte growth factor; SDF-1: stromal cell-derived factor-1; IGF-1: insulin-like growth factor-1; H/R: hypoxia/reoxygenation; c-kit: stem cell factor receptor; AMI: acute myocardial infarction.

### 2.6. Mechanisms Involved in Cardiac Regeneration

When stem cells where injected into damaged heart, they found a hostile microenvironment, that was characterized by a reduction of blood supply, deprivation of nutrients and hypoxic condition, secretion of cytokines with pro-inflammatory properties, such as TNF-α, IL-1β, and IL-6. In this context, an overproduction of free radicals determines an alteration of myocardial energy and metabolism, contributing to CM loss, pathological remodeling processes, promoting inflammation and cell death and reducing stem cell engraftment [142,143]. Accordingly, as mentioned above, albeit several stem cell types were employed to promote cardiac restoration, it is still necessary to develop new and safety approaches to be able to counteract cardiac cell death, hostile microenvironment, and promote stem cell proliferation and survival. To develop new methods to sustain stem cell therapy, it is important to have a better comprehension about the principal processes behind stem cell induced cardiac repair. On the other hand, heart development represents a dynamic process directed by a complex network of signals and gene transcription [144]. Some major signaling pathways are involved in the process from early cardiac tissue specification of mesoderm progenitors to subsequent differentiation into cardiac progenitors. These are the members of the TGF-β cytokine family, BMPs, Nodal/activin and the wnt/β-catenin pathway [73,90]. Moreover, a key role in heart development is played by paracrine signals responsible for the fine-tuning of those pathways [73].

In particular, a critical and most studied wnt pathway is the canonical signaling by the wnt family of secreted glycolipoproteins (hereafter wnt/β-catenin signaling pathway) which is an essential mechanism that direct cell proliferation and cell fate determination during embryonic development, adult homeostasis, tissue and organ regeneration, and specifically, is an important key factor in heart muscle development [73,74,75,76,77,78,79,80,81,82,83,84,85,86,87,88,89,90,91,92,93,94,95,96,97,98,99,100,101,102,103,104,105,106,107,108,109,110,111,112,113,114,115,116,117,118,119,120,121,122,123,124,125,126,127,128,129,130,131,132,133,134,135,136,137,138,139,140,141,142,143,144,145,146].

**Figure 2:**
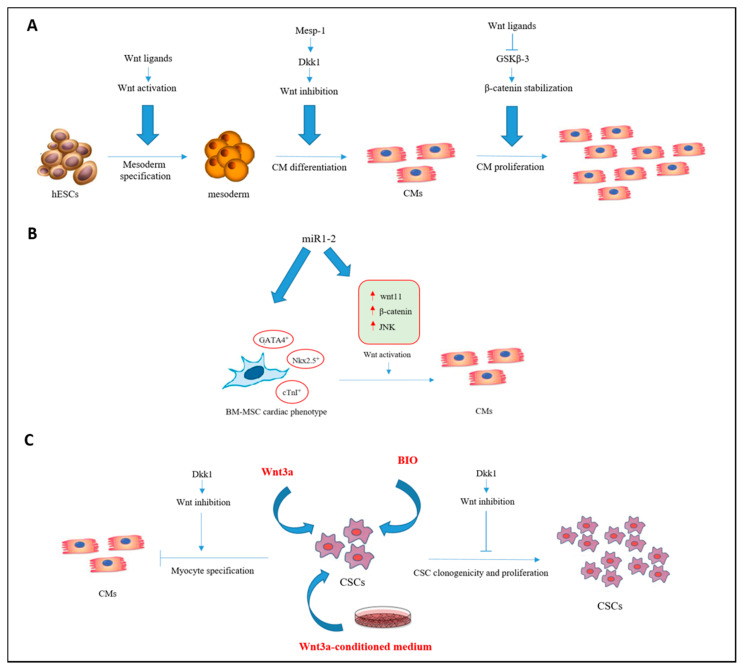
The biphasic role of wnt/β-catenin pathway in heart muscle development. (**A**) Wnt/β-catenin pathway promotes mesoderm specification in the early stages of hESC differentiation, while, its activation inhibits CM differentiation in the later stages. The cardiac differentiation inducer Mesp1 activates the inhibitor of canonical wnts, Dkk1, promoting CM differentiation. Moreover, the presence of wnt ligands inhibit GSK3β and lead to β-catenin stabilization in the cytoplasm and its traslocation into the nucleus promoting CM proliferation. (**B**) The overexpression of miR1-2 induces the expression of cardiac specific genes BM-MSCs and activates the wnt/β-catenin signaling pathway promoting their cardiac differentiation. (**C**) Wnt-3a, wnt-3a-conditioned medium and BIO stimulate CSC proliferation and clonogenicity, while, significantly inhibit CSC differentiation into myocyte-committed cells. In contrast, Dkk1 decreases CSC proliferation and clonogenicity and promotes myocyte specification, through the inhibition of the canonical wnt pathway. hESCs: human Embrionic Stem Cells; CMs: Cardiomyocytes; Dkk-1: Dickkopf-related protein 1; GSK3-β: Glycogen synthase kinase 3- β; BM-MSCs: Bone Marrow derived Mesenchimal Stem Cells; Nkx2.5: NK2 transcription factor related locus 5; cTnI: cardiac Troponin I; CSCs: Cardiac Stem Cells; JNK: c-Jun N-terminal kinase; BIO: bromoindirubin-3′-oxime; ↑ increase.

Studies in the past decades have identified wnt/β-catenin signaling in different physiological and pathophysiological processes, including angiogenesis, vasculature remodeling, myogenesis, adipogenesis, lipid and glucose metabolism and stem cell renewal and differentiation [147]. Several studies conducted on wnt/β-catenin pathway showed that it has biphasic role in heart muscle development: In the early phase of development, the activation of wnt/β-catenin pathway leads to cardiac specification, while in a late phase it inhibits it. However, its modulation can lead to different effects. For example, the treatment of ESCs with wnt ligands leads to CM formation through mesoderm specification, and a subsequent treatment with Dickkopf1 (Dkk1), a specific inhibitor of the wnt pathway, promotes CM differentiation (Figure 2) [148,149]. It was observed that the wnt pathway is also implicated in the differentiation of BMSCs through the specific regulatory activity of miR1-2 [150]. Indeed, the over-expression of miR1-2 in mouse BM-derived MSCs increases the expression of cardiac-specific genes Nkx2.5, cardiac Troponin I (cTnI) and GATA-4 which drive cells to a cardiac phenotype. In addition, miR1-2 activity leads to increased expression of wnt11, c-Jun N-terminal kinase (JNK), β-catenin and T-cell factor (TCF) in the wnt/β-catenin signaling pathway (Figure 2) [150]. However, this type of cells still lacks the CM morphology and did not beat unlike it has been observed in ESCs [151]. Moreover, in CSCs the presence of wnt ligand promotes clonogenicity and proliferation, while, the inhibition of canonical pathway of wnt, using Dkk1, promotes CM differentiation [152]. Despite some studies have suggested that the engraftment of stem cells leads to cardiac benefits, it was observed that the small number of cells engrafted cannot be the only responsible of this effect [153,154] (Figure 2). As previously introduced, the paracrine effect plays a pivotal role in the reparative action of stem cells after their injection into the damaged heart. Indeed, stem cells are able to release growth factors and/or chemokines that can promote cardiac repair in autocrine or paracrine manner [154]. The release of paracrine factors has been shown to be important for stem cell differentiation into CMs and in apoptosis reduction, thus, promoting the differentiation of resident CSCs and neovascularization. Moreover, anti-fibrotic, anti-oxidant and anti-inflammatory effects have been recognized, able to protect the microenvironment of the transplanted CSCs or resident CSCs [155,156]. Interestingly, it was observed that the use of preconditioned medium rich of VEGF, β-FGF, IL-1β, Platelet-Derived Growth Factor (PDGF), IGF-1, and TGF-β of BM-mononuclear cells, cultured in normoxic condition and amplified under hypoxic condition, improves cardiac function, reduces apoptosis of CMs and promotes angiogenesis [155,156]. Furthermore, Miao et al. clearly showed that the paracrine effects related to MSCs also determine an anti-inflammatory action, with a reduction of TNF-α, IL-6, and IL-1β, while, the release of VEGF and SDF-1 determine pro-angiogenic effects. It was also observed that the treatment with VEGF in combination with transplanted MSCs could increase vascular density, decrease the formation of scar tissue and improve cardiac function [124]. Moreover, the activation of endogenous CSCs through paracrine mechanism seems to be helpful, as it was recently reported. Injection of the growth factors IGF-1 and HGF in an animal model of AMI, evidently shown a regeneration of damaged myocardium, promoting proliferation and reducing fibrosis and CM reactive hypertrophy [140]. These and other evidence strongly support the idea that paracrine effects mainly promote cardioprotective actions and suggest that the use of growth factors can be useful for cardiac repair. In addition to soluble paracrine molecules, exosomes were proposed as mediators of adjacent or distant cells communication [157]. Immune-regulatory, proliferative and differentiation effects are ascribed to these vesicles due to the presence of proteins, lipids, mRNA, miRNAs [154]. They can function as signaling modulators, transferring membrane receptors and other proteins to the target cells, or modifying cell phenotype by the transfer of genetic information [158]. In particular, exosome properties were related to cardiac protection and regeneration [159]. Recently, it was shown that exogenous transplanted stem and progenitor cells can release exosomes, which were taken up by myocardial cells thus carrying out cardioprotective effects improving tissue microenvironment and boosting survival and engraftment of the transplanted cells [160]. Exosomes exert their cardioprotective activity through the delivery of mRNAs, miRNAs, and proteins to the injured heart muscle ameliorating resident cardiac stem cell activity, promoting CM proliferation, beneficial cardiac remodeling and angiogenesis. Several preliminary studies showed that exosomes released from different cell type, such as CPCs, MSCs or hiPSC-derived CMs are able to improve cardiac function through the modulation of specific gene expression, the stimulation of some metabolic processes and the reduction of oxidative stress, activating the Phosphoinositide 3-Kinases (PI3K)/Akt pathway and enhancing CM viability in different experimental model of damaged heart [47,161,162]. Moreover, cell precondition promotes exosome release [163] and increases the content of miRNAs within the exosomes resulting in a greater spread of cardioprotective signals within the myocardium [47]. Therefore, the study of stem cell-derived exosomes and their potential use as genetically engineered exosomes could be advantageous because these are cell-free component, have long-term stability, and low immune response. However, there are some limitations to their use, such as the need of repeated injections, target cell selection, and the random packing of the exosome cargo [164].

Based on the abovementioned mechanisms, researches proposed the use of growth factors, wnt modulators, genetic process or tissue engineering to increase cell viability, cell engraftment and reducing cell death to promote cardiac restoration. However, no safe and efficacy methods is actually available to support stem cells therapy in counteract cardiac remodeling, HF and cardiac dysfunction [75,140,165]. Interestingly, several studies reported below, have suggested that the use of natural compounds could be useful in stimulating stem cell survival, proliferation and differentiation, enhancing cell related paracrine effects and definitively cardiac repair.

## 3. Plant Extracts and Their Role in Nutraceutical Supplementation

The use of natural compounds in the treatment of several diseases dates back to ancient times around 3900 b.C. The Egyptians, Oakes and Gahlin, Greek, Chinese and Indian employed herbal remedies and wrote about their properties (for example in “The Ebers Papyrus”, “The Materia Medica” or “Pen T’Sao,”) handing down their use. The disclosure of possible therapeutic benefits of natural products traced back to 60,000 years ago. From that moment the knowledge about natural remedies began to pass from person to person, according to the characteristics of the different societies that continued to pass on and enrich it [165]. Traditional use of natural products includes several plants handled by different populations worldwide. For example, in the United States and in Northern Mexico, plant genus *Salvia*, was burned by Indian tribes to obtain hot ashes to cover the body of expectant mother during childbirth, while, in China the root of *Salvia miltiorrhiza* was used to dilate coronary arteries or as an antianginal drug, circulatory stimulant or sedative [166,167]. Ayurvedic therapies suggested the use of *Rauwolfia serpentine* root as a remedy for psychosis and hypertension, while, in the traditional Chinese medicine *Stephania tetandra* was used to treat hypertension, and the root of *Panax notoginseng* was employed in patients with angina and coronary artery diseases [166]. Furthermore, plants of the genus *Mercurialis spp*. were widely used in Spain to different aims depending on the species of Mercurialis (*M. annua, M. ambigua, M. perennis, M. tomentosa):* It can be used as an anti-hypertensive, laxative, abortive, anti-hyperglycemic, anti-arthrosis, hepatoprotective or anticolagogue [168]. In the 18th century, thanks to “Systema Naturae” (1735) and “Species Planetarium”, from Carolus Linnaeus, the botanical classification and the identification of thousands plants with specific characteristics began. These works represented a fundamental and useful resource for botanists and taxonomists in the drafting of subsequent collections about traditional herbal remedies, described by anthropologies and ethnobotanicals in several cultures [169], and which represent important sources for the discovery of new natural drugs in the field of scientific research [170]. The historical use of plants for health care has come down to the present day thanks to the beneficial effects in the treatment of pain, inflammation, microbial infection or gastrointestinal diseases observed in several experimental studies [171]. In particular, it was found that the beneficial effects of plants include their ability to counteract congestive HF, systolic hypertension, angina pectoris, atherosclerosis, venous insufficiency and arrhythmia, probably due to the presence of several biomolecules acting through different mechanisms [171]. The most common examples are cardioactive glycosides, present in *Strophanthus hispidus* and *Strophanthus kombe, Thevetia peruviana* and *Urginea maritima*, which have positive inotropic actions. Even the biomolecules digitoxin in *Digitalis purpurea*, or digoxin in *Digitalis lanata* are glicosides responsible for the beneficial effects in different heart conditions and used in case of congestive HF [166,169]. In addition, flavonoids, oleuropein, ω-3 fatty acids, alkaloids and lycopene are natural compounds found in several plants, which can be useful in the prevention and management of CVDs as has been observed in many studies. The beneficial effects of all these natural compounds are mainly linked to their antioxidant, anti-inflammatory, antiplatelet, anti-hypercholesterolemic, anti-hypertensive actions, and to their ability to modulate High Density Lipoprotein (HDL)/Low Density Lipoprotein (LDL) ratio, protecting the endothelial function [164,165,166,167,168,169,170,171,172,173,174]. All these characteristics suggest that natural compounds could potentially be beneficial in counteracting the main risk factors and early pathological features of CVDs contributing to cardiac recovery. The study on the synergistic modulation of natural compounds and stem cells in counteracting cardiac damage highlights the key role played by some of them.

### Natural Compounds and Cardiovascular Protection

In this review, we focus on lupinine, resveratrol, ginseng components, icariin, curcumin and BPF. Recent data interestingly showed how these compounds met the requirements for the activation, proliferation, differentiation and survival of stem cells that promote cardiac repair, suggesting a possible combined use of natural cardioprotective agents with stem cell-based therapy, as will be discussed in the next section.

Concurrently, the safety profile and specific cardioprotective properties of these natural compounds have been extensively described in several experimental models and clinical trials of CVDs such as HF, cardiac ischaemia and myocardial fibrosis.

In brief, lupine belongs to the quinolizide family and is one of the major alkaloids present in the seeds of *Lupinus luteus* and other various lupine species (*Lupinus caudatus* L., *Lupinus albus* L.) [175]. Although there are few studies regarding the possible beneficial effects of lupine against CVDs, some reports have shown specific hypoglycemic properties of lupine extracts leading to improved glucose homeostasis in glucose-resistant experimental models [175,176]. Moreover, interesting cardiovascular health benefits, in terms of insulin sensitivity and blood pressure were observed in a randomized controlled weight loss trial of overweight individuals treated with a lupine-enriched diet [177].

The major bioactive compound of horny goat weed is icariin, also known as Epimedii Herba or Ying Yang Huo [178,179] which represents the principal pharmacologically active constituent in *E. brevicornu Maxim*, *E. koreanum Nakai*, *E. sagittatim Maxim*, *E. pubescens Maxim* and *E. wushanense* T. S. Ying [179]. Different studies clearly shown that icariin exerts a strong anti-oxidative, anti-inflammatory, and lipid-modulatory effects, preventing the development of cardiovascular risk factors. Indeed, icariin has protective effects against free-radical-induced peroxidation [180] and has shown anti-oxidant properties after a pre-treatment in cardiac myocytes, reducing cell apoptosis, preventing mitochondria membrane potential and preserving Ca^2+^ homeostasis, increasing superoxide dismutase (SOD) activity and reducing malondialdehyde (MDA) levels. These effects were related to a direct reactive oxygen species (ROS) scavenge action and to the up-regulation of p-ERK exerted by icariin [181]. Icariin is able to suppress the expression of TNFα, *cyclooxygenase-2* (COX2), inducible NOS, and *myeloperoxidase* (MPO) activity through the inhibition p38/MAPK and NF-κB p65 pathways [182]. Icariin also showed specific atherosclerotic activity, improving the lipid profile in experimental models of atherosclerosis [183]. It also suppresses the oxidized LDL formation, leading to anti-proliferative effects in VSMCs, and mediates nitric oxide (NO) production and vasorelaxation, thus, preventing endothelial dysfunction through the activation of PI3K/pAkt/p-eNOS pathway in several animal models of atherosclerotic diseases [178,184]. Specifically, icariin increases endothelial Nitric Oxide Synthase (eNOS) level and NO production through the activation of PI3K/pAkt/p-eNOS pathway and ERK pathway, reducing eNOS uncoupling thanks to the inhibition of ROS levels [184]. The cardioprotective properties of icariin and its potential involvement in the modulation of the PI3K/Akt signaling pathway were also observed in different animal models of ischemia-reperfusion injury and cardiomyopathies. Indeed, icariin perfusion before ischemia induction and throughout reperfusion, ameliorates cardiac function improving left ventricular diastolic pressure, coronary flow and reducing infarct size, oxidative stress and cardiomyocyte apoptosis [178,185,186,187]. Moreover, icariin preserves the injured heart from pathological cardiac remodeling, characterized by hypertrophy and degeneration of CMs and inflammatory infiltration, down-regulating Matrix Metallopeptidases (MMP)-2 and MMP-9, increasing Bcl2 levels and decreasing Bax and caspase 3 [187].

Ginseng is a perennial umbel plant and belongs to the genus *Panax*, commonly referred to the dry root and rhizome of *Panax ginseng* C.A. Meyer of the family Araliaceae [188,189]. Traditionally, Ginseng was considered a panacea in promoting longevity, and its use as a tonic was considered helpful for mind and fatigue, also useful to increase physical strength, to prevent aging, to increase vigor, and was reported in many herbals which led to counteract several illnesses in East Asian countries [188,189]. All the positive actions ascribed to this plant, bring to the study of the active biomolecules of ginseng, leading to the isolation of ginsenosides, alkaloids, phenolics, phytosterol, polypeptides, ginseng oils, nitrogenous substances and vitamins [188]. Ginsenosides are triterpene saponins, which represent the main bioactive metabolites of ginseng are divided into the panaxadiol group (Rb1, Rb2, Rb3, Rc, Rd, Rg3, Rh2, Rs1) and the panaxatriol group, (Re, Rf, Rg1, Rg2, Rh1), and account for ginseng beneficial effects in several pathological conditions, such as CVDs, cancer and immune deficiency [190]. The cardiovascular benefits of ginseng and ginsenoides are mainly attributable to their antioxidant properties but interesting anticoagulant, antihypertensive antihyperglycaemic and hyperlipemic effects are also observed in several studies [189]. The antioxidant activity of ginseng specifically concerns the reduction of MDA levels and the increase in endogenous antioxidant enzymes concentration, such as SOD, CAT, and GPx. The administration of ginseng water extract exhibits a significant reduction in oxidative stress, downregulating ROS-stimulated mitogen-activated protein kinase (MAPK) and Akt pathways in in vitro and in vivo models [188,190,191]. The antioxidant activity of ginseng is also mediated by the activation of Nrf2, a transcription factor of endogenous anti-oxidative defence systems [192]. In addition, other several studies have shown that total ginsenosides activate PI3K, eNOS and Akt pathway, determining an increase in NO levels and a subsequent improvement of vascular function and platelet aggregation rate [193,194]. Moreover, pre-treatment with ginseng was found able to counteract pathological electrocardiogram (ECG) abnormalities and changes in left ventricular systolic pressure in a rat model of isoproterenol-induced cardiac injury through the increase of myocardial antioxidative defense system and the inhibition of neutrophil infiltration in cardiac tissue [195,196]. In particular, ginsenoside Rg3, when administrated in a rat model of myocardial ischemia reperfusion, improves cardiac function and decreases left ventricular diastolic pressure, inhibiting caspase-3, p53 and reducing left ventricle TNF-α and IL-1β levels [196]. Interestingly, the treatment with ginseng extract for 8 months in ST-elevation AMI patients, after coronary stenting, reduces inflammatory cytokines (IL-6, TNFα, VCAM-1) production, increases circulating angiogenic cells ameliorating the recovery of microvascular integrity [197]. Several other randomized clinical trials reported clear pharmacological and medical applications of ginseng and gingenosides, resulting in beneficial effects on cardiac and vascular diseases through the improvement of lipid profiles, the adjustment of blood pressure, the enhancement of cardiac function and the reduction in platelet adhesion [189,198]. Another extensively studied natural antioxidant compound is curcumin, a diferuloylmethane which represents the major natural polyphenol derived from the rizoma of turmeric, *Curcuma longa* and Curcuma spp. [199,200]. The protective role of curcumin in CVDs is related to its antioxidant and anti-inflammatory properties associated with the chemotherapeutic effects and, in particular, the anti-thrombotic, and cardioprotective action [201,202]. The antioxidant effect of curcumin seems to be mediated by Nrf2, which interacts with antioxidant response elements by inducing the transcription of antioxidant enzymes, while, its anti-inflammatory properties are mediated by the activation of heme oxigenase (HO-1) [203]. Several studies identified curcumin as a remedy to counteract many cardiovascular pathological features such as aortic aneurysm, atherosclerosis, cardiac hypertrophy, drug-induced cardiotoxicity, diabetic complications [201]. Based on its antioxidant activity curcumin inhibits lipid peroxidation, oxidized LDL formation and inflammation. Curcumin also reduces the total cholesterol content into foam cells, preventing smooth muscle cells migration and proliferation and monocyte adhesion [204]. Interestingly, curcumin has shown a suppressive action in several experimental model of cardiac hypertrophy and HF, mainly due to its well-known inhibitory activity on the transcriptional co-activator p-300 involved in the pathological growth of CMs and on the interaction between p300 and the key transcription factor GATA-4 [205]. Curcumin significantly prevents apoptotic cell death, reducing infarct size, inflammatory response and pathological cardiac remodeling in different animal models of MI [206]. The authors suggested that the cardioprotective effect is due to the ability of curcumin to modulate the PI3K/Akt, ERK1/2 and GSK3β pathways [207]. Its antioxidant and anti-apoptotic properties lead to the phosphorylation of JAK2 and STAT3, enhancing Bcl-2/Bax expression and reducing caspase-3 [208]. Also noteworthy is Resveratrol, a proanthocyanidins, specifically hydroxy stilbenes, derived from grapes, plumps, blueberries red wine with powerful antioxidant and anti-inflammatory properties [209]. The influence on NO availability and the antioxidant effect explain the beneficial effects of resveratrol on CVDs. Indeed, resveratrol significantly increases NO levels through the upregulation of eNOS and VEGF levels, preventing endothelial dysfunction in a rat model of myocardial ischaemia [210]. Resveratrol has been shown to increase the endogenous antioxidant protective mechanism in the heart by reducing free radical species through inhibition of pro-apoptotic JNK and c-fos proteins, after myocardial ischaemic/reperfusion injury [210]. Moreover, resveratrol is able to reduce oxidative stress by enhancing MnSOD expression and glutathione levels, decreasing stress markers (MAPK p38, ERk1/2) and increasing prosurvival marker phosphor-Akt and GSK3β [211]. In addition, pre-treatment with resveratrol is able to reduce cardiac hypertrophy and fibrosis, decrease infarct size in several experimental models of HF thus restoring left ventricular function [212]. Several studies reported clearly antihypertensive properties of resveratrol, able to reduce blood pressure in different animal models of myocardial ischaemia with potential regeneration capacity in the affected area, maybe due to the activation of Akt and Bcl2 and of the autophagic pathway [213]. Interestingly, patients with stable coronary artery disease showed an improved diastolic function after resveratrol treatment [214]. Moreover, experimental evidence exists about the ability of resveratrol to decrease LDL-cholesterol, improve systolic function and endothelial function, and decrease platelet aggregation in post-infarction patients [215]. Among all, bergamot polyphenolic fraction (BPF), derived from *Citrus bergamia* Risso et Poiteau, shows potential protective activities in the management of different features of atherosclerosis, metabolic disorders, and cardiotoxicity due to its pleiotropic anti-oxidative, anti-inflammatory and lipid-lowering effects [216]. In the last decades, several in depth studies, performed on cellular and animal models, and clinical trials demonstrated hypolipemic and anti-atherogenic effects of BPF, associated with the modulation of the activity of some enzymes responsible for cholesterol esterification reactions and lipid trafficking [217,218]. Moreover, some of these polyphenols inhibit the rate-limiting step in cholesterol synthesis due to structural similarity to the HMGCoA reductase substrate [219]. Furthermore, BPF is able to interfere with the autophagic pathway preventing the pathogenic lipid accumulation and strongly induce lipophagy, as shown in a rat model of metabolic syndrome [220]. In particular, it was recently observed that BPF directly induces the modulation of JNK/p38 MAPKs, ameliorating insulin sensitivity in animal models of metabolic syndrome and pathological fatty liver [221]. The powerful antioxidant effects of BPF underlie all the observed protective effects. BPF directly reduces lipid peroxidation biomarkers (TBARS), MDA, strongly inhibits protein tyrosine nitration levels and prevents ROS accumulation in the nucleus of several cell types [222,223,224]. BPF also improves the activity of endogenous antioxidant enzymes, such as SOD, Glutathione Peroxidase (GPx) and Glutathione S Transferase P1 (GSTP1) [225,226]. The additive vaso-protective effect of BPF, related to its antioxidant property, demonstrated its ability to reduce oxidized low-density lipoprotein receptor-1 (LOX-1) expression levels, highly modulated in the development and progression of endothelial dysfunction to atherosclerosis, and increase PKB phosphorylation, providing protection against vascular atherogenic injury in patient with hyperlipemia [222]. Interestingly, BPF exerts cardioprotective effects suppressing ROS production, excessive autophagy activation and apoptosis of CMs and resident endogenous CSC compartment in an experimental model of doxorubicin-induced acute cardiomyopathy, thereby, attenuating pathological cardiac remodeling and left ventricular dysfunction [223]. In the light of these experimental evidence about the beneficial cardioprotective effects of compounds deriving from natural products these may represent an innovative tool in supporting stem cell therapy looking for new safety and efficacy approaches in cardiac restoration.

## 4. Natural Compounds and Stem Cells Activation and Differentiation in CVDs

**Figure 3:**
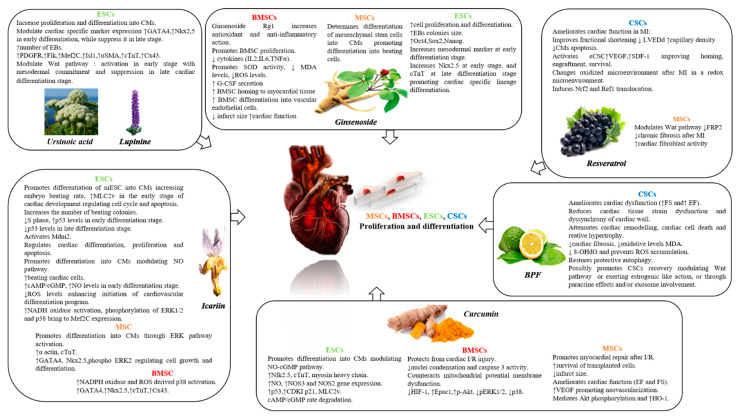
The role of certain natural compounds in stem cell activation. ↑ increase, ↓decrease. ESCs: embryonic stem cells; CMs: cardiomyocytes; GATA-4: GATA-binding protein 4; Nkx2.5: NK2 transcription factor related locus 5; EBs: embryoid bodies; PDGFR: Platelet-Derived Growth Factor Receptor; Mef2C: Myocyte Enhancer Factor 2c; Isl1: Insulin gene enhancer protein-1; α-SMA: α-Smooth Muscle Actin; cTnI: cardiac Troponin I; Cx43: Connexin 43; BMSCs: bone marrow stem cells; MSCs: mesenchymal stem cells; IL-2: interleukin-2; IL-6: interleukin-6; TNF-α: tumor necrosis factor-α; SOD: superoxide dismutase; MDA: malondialdehyde; ROS: reactive oxygen species; G-CSF: granulocyte colony-stimulating factor; Oct4: Octamer-binding transcription factor 4; Sox-2: SRY-box transcription factor-2; CSCs: cardiac stem cells; MI: myocardial infarction; LVEDd: left ventricular end diastolic diameter; VEGF: vascular endothelial growth factor; SDF-1: stromal cell-derived factor-1; MLC2v: myosin light chain 2v; Mdm2: E3 ubiquitin-protein ligase; NO: nitric oxide; FS: fractional shortening; EF: ejection fraction; NOS3: nitric oxide synthase 3; NOS2: nitric oxide synthase 2; CDKI p21: cyclin-dependent kinases inhibitor p21; HIF-1 α: Hypoxia-Inducible Factor-1α; I/R: ischemia/reperfusion; HO-1: heme oxigenase-1.

Although limited data is available on the role of nutraceuticals in the modulation of stem cell properties, it is interesting to note how the targeted use of certain natural occurring compounds can contribute to the activation, proliferation and differentiation of different types of stem cells, intensifying their protective activity against cardiovascular damage. The most interesting and recent results obtained from the analysis of the mainly studied natural compounds are discussed (Figure 3).

### 4.1. Lupinine and Ursinoic Acid

As mentioned above, lupinine, represents the main alkaloid in the seeds of *Lupinus luteus* and other various species of lupine (*Lupinus caudatus* L., *Lupinus albus* L.) [175], while ursinoic acid is an aromatic oxo acid isolated from the roots of Angelica ursina but it is not pointed out for any therapeutic application [226]. However, an in-depth study recently shown that the concomitant use of these two natural compounds promotes the commitment of pluripotent stem cells to cardiac mesoderm and contribute to CM differentiation [227]. Lupinine and ursinoic acid were used to treat mouse embryonic CSCs and mouse embryo-derived teratocarcinoma cells (P19 cells). In particular, a CM-specific readout reporter system based on the P19 cell line (which has a pluripotent nature) was used expressing the α-myosin heavy chain (α-MHC) promoter-driven fluorescent mCherry protein, which is activated upon cardiac induction. After in vitro formation of EBs from both cell lines, P19 and mouse embryonic CSCs, were treated for three days with or without natural product library containing 800 compounds of various alkaloids, flavonoids, and sterols to initiate differentiation. At the end of the treatment, the cells continued their differentiation under attachment conditions for another three days and then were observed for mCherry expression. Among a number of natural compounds, lupinine and ursinoic acid successfully induced a consistent improvement in the fluorescence of mCherry and 0.5 mM of lupinine and 0.25 mM of ursinoic acid concentrations were chosen for subsequent experiments [227]. Treatment of cells with these two natural compounds showed higher differentiation efficiency when compared to the untreated cells or cells treated with oxytocin, used as a positive control. In particular, the rate of differentiation was observed by the increase in the number and size of the beating colonies in lupinine and ursinoic acid treated cells. Analysis of the transcript levels of early cardiac-specific differentiation markers, GATA-4 and Nkx2.5, recognized an increase in both P19 and mouse embryonic CSCs, which gradually increase up to 10 days. After 10 days the levels of these cardiac markers decrease, suggesting that cells treated with natural compounds proceed to the next stage of maturation where these markers were not relevant. In addition, lupinine and ursinoic acid are involved in cardiogenesis has demonstrated by the upregulation of PDGFR-α and Flk, cardiac progenitor cell markers involved in the middle stage of cardiac differentiation. Furthermore, the combined use of these two natural compounds also increases the Myocyte Enhancer Factor 2c (MEF2C) and the Insulin *gene* enhancer protein (Isl), markers of middle and early stages of cardiac differentiation. Muscle and cardiac markers of the most differentiated cells (α-Smooth Muscle Actin-α-SMA and cTnT) were also increased after treatment of both lupinine and ursinoic acid proving the efficacy of differentiation. Levels of α-SMA, cTnT and Connexin 43 (Cx43) were also increased in mouse embryonic stem cells (mESC) the levels of [227]. Interestingly, the improvement in cardiogenesis due to ursinoic acid and lupinine treatment was associated with the activation of the wnt pathway. In particular, an upregulation of the wnt pathway in EBs was observed after 24 h of treatment with these two alkaloids, which was suppressed after 72 h. These data suggest that lupinine and ursinoic acid promote the activation of the wnt pathway during the early phase of EBs, contributing to an efficient cardiac mesoderm commitment and subsequently their effects decrease, leading to the transition from mesoderm to the next stage of cardiac commitment [227].

### 4.2. Resveratrol

The properties of resveratrol in stem cell therapy has been demonstrated in several experimental studies. Among the reported results, it was observed that the administration of 2.5 mg/Kg of resveratrol can improve cardiac regeneration through the activation of endogenous CSCs before and after the induction of AMI, together with the injection of CSCs into the peri-ischemic area [228]. In this experimental model, resveratrol improves cardiac function ameliorating FS and reducing left ventricular-end diastolic diameter. This effect is linked to an increase in capillary density, decreased CM apoptosis and upregulation of VEGF and SDF-1, suggesting that the activation of endogenous CSCs could enhance cardiac recovery [229]. Furthermore, resveratrol improves the homing, engraftment and survival of injected CSCs in an animal model of MI. In particular, the CSCs were preconditioned with 2.5 uM of resveratrol before being injected in the border zone of ischemia [230]. Interestingly, improved cardiac function and a modification of the microenvironment was observed, as demonstrated by the nuclear translocation of Nrf2. It binds to the antioxidant response element and to Ref-1, a redox protein that regulates redox-sensitive transcription factors, leading to an increased engraftment of the implanted stem cells [230]. The ability of resveratrol to improve stem cell survival and engraftment was confirmed by the expression of the cell proliferation marker Ki67, while the expression of SDF-1 and myosin clearly demonstrated CSC homing in the infarcted myocardium and its regeneration which ameliorates cardiac function [230]. These results showed that resveratrol is able to modify the oxidized microenvironment after MI in a redox setting for CSCs, exerting an interesting beneficial effect in cardiac restoration [228,230]. In another in-depth study, intraperitoneal injection of 2.5 mg/kg of resveratrol and a tail vein injection of conditioning MSCs with 0.1 µM resveratrol were performed [231]. Resveratrol has been shown to reduce sFRP2, a key regulator of the wnt pathway. sFRP2 binds to Fz receptors and wnt ligands, playing a key role in chronic fibrosis after MI and may enhance the activity of adult mouse cardiac fibroblasts through wnt/β-catenin signaling. The observed effects are related to an improvement in EF, as well as a reduction of cardiac remodeling and cardiac fibrosis [231].

Moreover, resveratrol is able to promote mesoderm differentiation of human iPSCs under EB condition, up-regulating the expression of the Mesoderm Posterior BHLH Transcription Factor 1 (MESP1), Brachyury, and Mix Paired-Like Homeobox-1 (Mxl1) markers. In addition, the rate of beating EBs, after resveratrol treatment, was higher than in the control group. Resveratrol significantly reduces Oct4 and Nanog expression and substantially enhances the expression of specific cardiac genes Nkx2.5, GATA-4, cTnT, α-Myosin Heavy Chain (α-MHC), thus promoting CM differentiation of human iPSCs [232]. An interesting in vitro study using mouse ESCs identified the optimal concentration of resveratrol (10 umol/L) capable of directing mouse ESC differentiation into CMs and of achieving the beating properties of EBs [233]. Notably, the authors reported significantly higher levels of the five cardiac gene markers analyzed, including Nkx2.5, MEF2C, Tbx5, dHand and α-MHC, in mouse ESC treated with 10 umol/L of resveratrol compared to other concentrations. Additionally, the highest protein levels of Cx43 and Troponin C1, maturity indicative cardiac markers, were observed. These results were also associated with the largest calculated beating areas [233]. The mechanism underlying these properties of resveratrol seems to be related to the inhibition of the canonical wnt pathway, and upregulation of SRF/mir-1 expression, both of which are involved in CM commitment from human cardiovascular progenitors [232,233]. In an animal model of cardiomyopathy resulting from doxorubicin-induced cardiotoxicity the intriguing effect of resveratrol, associated with adipose-derived MSCs was investigated [234]. The data showed that the injection of resveratrol and adipose-derived MSCs significantly prevent the onset and severity of cardiotoxicity induced by concomitant treatment with doxorubicin. Animals treated with both resveratrol and adipose-derived MSCs showed no signs of cardiac necrosis or edema, while myocardial healing consisted of myofibril regeneration was observed. Furthermore, cell cluster of adipose-derived MSCs were observed suggesting that differentiation of adipose-derived MSCs into CMs associated with MSC paracrine capacity contributes to cardiac improvement against doxorubicin detrimental effects [234]. In addition to the reported data, MSCs previously treated with resveratrol and injected into a failed myocardium, induce cardiac differentiation and enhance MSC paracrine effects in an animal model of doxorubicin-induced cardiomyopathy [235].

### 4.3. Ginseng

Although there is few information about ginseng treatment and stem cell differentiation into CMs, an interesting report has shown that the use of ginsenoside Rg1 improves the antioxidant and anti-inflammatory capacities of BMSCs in the hematopoietic microenvironment of aged rats [236]. Aging is a condition characterized by chronic inflammation, increased levels of ROS and decreased levels of anti-oxidant defense. These conditions were reverted by the use of ginsenoside Rg1, able to counteract the increase in inflammatory cytokines (IL-2, IL-6, TNF-α), MDA content and ROS levels and promote SOD activity, leading to BMSC proliferation [236]. As mentioned above, one of the mechanism through which ginseng determines cardioprotection is the upregulation of NO. Indeed, ginseng is an angiogenesis inducer of eNOS expression thanks to the ginsenoside Rg1. Rg1 was found to decrease miR-214, which appears to be involved in the negative modulation of eNOS expression in human umbilical vein endothelial cells (HUVECs), suggesting a possible angiogenic mechanism induced by ginsenoside [237]. In particular, BMSC-derived exosomes have been observed to strongly suppress apoptosis and ROS production in CSCs after oxidative stress injury, mainly due to the action of mir-214 in silencing Ca^2+^/calmodulin-dependent protein kinase II (CaMKII) target [238]. However, there are some studies that determined the action of ginseng on stem cells. Ginseng extract has been observed to facilitate proliferation and differentiation of hESCs [239]. In brief, ginseng extract was added to undifferentiated hESCs and differentiating cells at 0.125, 0.25, and 0.5 mg/mL. Human-ESC colonies were cultured in suspension for several days, while undifferentiated hESCs were treated to differentiate them into cardiac progenitor cells. At the end of the experimental period it was shown that ginseng-treated hESCs had larger colony size and increased proliferation rate and expression of pluripotency markers such as Oct4, Sox2, and Nanog. Ginseng also allows differentiation into mesendoderm lineage when added to EBs for 10 days, showing increased expression of the mesodermal marker (Brachyury and winged helix transcription factor hepatocyte nuclear factor 3β (*HNF3β* or *Foxa2*). Moreover, ginseng treatment promotes mesodermal lineage in the early stage of hESC-derived cardiac progenitor cell differentiation, while, it promotes cardiac specific lineage differentiation in the later stage of hESC-derived cardiac progenitor cells as showed by increased levels of Nkx2.5 and cTnI [239]. Interestingly, some of the compounds contained in the water soluble fraction of the ginseng fraction (in particular vitamin B12 and methionine) are able to promote the differentiation of MSCs into CMs, enhancing the differentiation rate into beating cells [240]. Finally, ginsenoside-Rg-1 was identified as a potent proangiogenic agent. Indeed, rg-1 has been shown to reduce miR-15b expression, leading to a temporal induction of VEGFR-2 in HUVEC cells. It has also demonstrated that rg1 induces motility and tubulogenesis through the upregulation of VEGFR-2 in treated cells. Confirming this result, in an in vivo model of zebrafish embryos, injection of the pre-miR-15b precursor was found to significantly suppress sub-intestinal vessels formation [241]. Other in vivo experiments also reported that Rg1 stimulates the myocardial tissue secretion of granulocyte colony-stimulating factor (G-CSF), able to promotes BMSC homing to myocardial tissue and differentiation into vascular endothelial cells. Interestingly, a reduction in the size of the myocardial infarction and an improvement in cardiac function were observed in this condition [242]. In a rat model of acute MI it was also observed how the continuous application of Rg1 enhances the number of peripheral blood CD34^+^ stem cells and stimulates the homing of stem cells to the infarcted area, leading to ventricular remodeling with reduction of infarct size and significant myocardial regeneration [243]. A detailed study recently published in Nature has identified the 20(R)-ginsenoside Rh_2_ among several ginseng saponins, capable of increasing the proliferation of skeletal myoblasts in vitro [244]. The molecular mechanism underlying this important effect was identified in the activation of Akt signaling and in the inhibition of the cyclin-dependent kinases inhibitors (CDKIs), p27^Kip1^ and p57^Kip2^ which negatively regulate cell proliferation. Furthermore, an animal model of MI and an animal model of skeletal muscle degeneration were used to assess the potential beneficial effect of ginsenoside Rh_2_ in vivo. The results showed that treatment with ginsenoside Rh_2_ increases cardiac recovery through improved left ventricular function and increased proliferation of CMs in the infarcted area which was indicated by higher levels of cTnI and Ki67. Additionally, in the animal model of barium chloride-induced skeletal muscle degeneration, treatment with ginsenoside Rh_2_ leads to a reduction in muscle scarring and fewer inflammatory cells. These features are associated with reduced tissue degeneration and a greater proportion of smaller diameter fibers, which increase in diameter as the muscle heals [244].

### 4.4. Icariin

Interesting data are collected about the effects of icariin in the modulation of cardiomyogenesis and in differentiation into CMs through several specific actions. An an in vitro study showed the ability of icariin to modulate cardiomyogenesis and promote differentiation of mouse ESCs into CMs [245]. Briefly, ESCs were cultured to form EBs and then plated on a gelatin-coated plate and treated with icariin 1×10^−7^ mol/L for 23 days. The rate of beating EBs was increased compared to control and cardiac differentiation process was improved. Indeed, an increase in cardiac-specific protein such as mRNA level of cardiac α-MHC and myosin light chain 2v (MLC2v) was observed in EBs in the early stage of cardiac development. Furthermore, the inducible effect of icariin was related to cell cycle regulation and induction of apoptosis as a signal of ESC differentiation [245]. In addition, treatment of ESC derived-embryoid stem cells with 10^−7^ M icariin for 24–48 h confirmed its ability to promote differentiation into beating colonies increasing the levels of cardiac specific markers. Moreover, in the early phase of differentiation, icariin induces cell cycle arrest and apoptosis, reducing the S phase in ESCs before transitioning to the CM phenotype with an increase in p53 levels. However, during the last phase of differentiation, a reduction in p53 protein expression levels, accompanied by the activation of the E3 ubiquitin-protein ligase (Mdm2), was observed, suggesting a potential mechanism by which icariin could regulate cardiac differentiation, proliferation and apoptosis [246]. Subsequent studies suggested that icariin mediates the differentiation of ESCs into CMs through modulation of the endogenous NO signaling pathway [247]. After EB induction, cells were treated with 1×10^−7^ mol/L icariin. The data showed that, in accordance with the study mentioned above, icariin increases beating cardiac cells expressing α-actinin and cardiac transcription factors. In particular, levels of GATA-4 and Nkx2.5 were increased by icariin treatment during early cardiac differentiation, as well as, the levels of α-MHC, MLC2v and β-adrenergic receptor (β-AR) [247]. Cardiomyocytes treated with icariin were shown to be more sensitive to isoproterenol than control cells. Furthermore, after 24–48 h of icariin treatment the ratio of cAMP/cGMP and NO levels was increased, before ESCs became CMs. Instead, the cGMP level was increased within 24 h, suggesting that the NO-cGMP pathway is mainly involved in the early differentiation phase of icariin treated ESCs. Therefore, the upregulation of NO production appears to be a mechanism by which icariin modulates the early differentiation of ESCs into CMs as has been observed using NO inhibitors which leads to a reduction in the differentiation process [247]. Interestingly, modulation of ROS levels was involved in icariin induced ESC-differentiation into CMs. Low levels of ROS during the early phase of differentiation of ESCs have been observed to be helpful in initiating the cardiovascular differentiation program [248]. The in vitro model of mouse ESCs-derived EBs treated with 100 nM icariin, paradoxically, showed that icariin (and other prenylflavonoids such as icaritin and desmetilicaritiin) was able to increase the number of spontaneous beating EBs, cardiac specific genes and cardiac transcription factors by regulating the intracellular concentration of ROS [248]. In particular, the effects of icariin are related to the activation of NADPH oxidase, to the phosphorylation of ERK1/2 and p38, bringing to MEF2C expression and CM differentiation [248]. Other reports support the theory that icariin promotes cardiac differentiation in BM-MSCs enhancing NADPH oxidase and ROS-derived p-38 activation. Co-administration of icariin (0.1 μM) and the BMP-2 (10 μg/L) for 24 h has been observed to exert a synergistic effect in CM differentiation of BM-MSCs in vitro, enhancing the expression of specific markers such as GATA-4, Nkx2.5, cTnT, and Cx43, higher levels of NADPH oxidase, H_2_O_2_ and p38MAPK [249]. Icariin was also found to induce CM differentiation in MSCs derived from adipose tissue and this effect was related to the activation of the ERK pathway [250]. Adipose-derived MSCs co-cultured with CMs showed increased protein levels of α-actinin and cTnT at 21 days after treatment with 10^−7^ mol/l icariin for 24 h, increasing the rate of differentiated cells compared to non-treated cells. In addition, GATA-4, Nkx2.5, MLC-2v markers increased after 3 weeks of treatment. These effects were supported by increased levels of p-ERK-1 and p-ERK-2, a general regulator of cell growth and differentiation in response to mitogenic stimuli [250].

### 4.5. Curcumin

Several studies reported a powerful effect of curcumin on histone acetylation, which can be useful in counteracting pathological cardiac growth, detrimental remodeling or HF. Indeed, cardiac growth and gene expression are regulated through histone acetylation/deacetylation in response to acute and chronic stress [251]. Recently, curcumin has been found to modulate cardiogenesis through inhibition of histone acetylation. In an experimental model, H9c2 cells were transfected with BMP-2 to induce cardiac specific gene expression of GATA-4 and MEF2C through p300 activation, thereby, increasing the level of H3 histone acetylation. Interestingly, this effect was reversed by curcumin’s inhibition of p300 [252]. An in-depth study investigated the key role of curcumin in epigenetic modifications of embryonic heart development such as in prenatal alcohol exposure model [253]. Indeed, in cardiac progenitor cells, curcumin inhibits alcohol-induced hyperacetylation of hystone H3, leading to a reduction in the expression of GATA-4 and MEF2C and subsequent abnormal heart development and congenital heart disease [253]. In addition to the above data, some studies have shown that pre-treatment of adipose MSCs with curcumin promotes myocardial repair in an animal model of cardiac ischemic-reperfusion injury, performed by left coronary artery ligation (LAD) [254]. One week after ligation, adipose MSCs treated with 10 μM of curcumin for 24 h were injected into the peri-infarcted regions and within the infarcted area. After 28 days, an increase in the number of vital transplanted cells was observed, accompanied by a reduction in the infarct size and an improvement in cardiac function with an increase in the level of VEGF. Furthermore, in vitro data showed that curcumin protects stem cells from H_2_O_2_ and reduces apoptosis by mediating Akt phosphorylation, increasing HO-1 expression and inhibiting phosphatase and tensin homolog (PTEN), p53, and caspase-3 expression levels [254]. Curcumin has also been found to modulate ESCs differentiation into cardiac lineage through the modulation of NO-cyclic GMP pathway [255]. Human-ESCs were cultured in suspension to form EBs and then transferred to coated plates with differentiation medium. EBs at day 1 and 2 and partially differentiated cells at day seven, were treated with curcumin (5–20 μmol/L) at day 7, 9, 11 and 13. At the end of the experimental period, increased levels of cardiac specific markers (NKx2.5, cTnI and α-MHC) determined by curcumin were observed. Moreover, an increase in intracellular nitrite, induction of the NOS-3 gene and reduction of NOS-2 was recorded alongside an increase in the expression of the soluble Guanylyl Cyclase α1 (sGC α1) gene, suggesting that NO pathway is involved in curcumin-induced stem cell differentiation [255]. Co-administration of curcumin with NO donors leads to an increase in the protein levels of p53, cyclin-dependent kinase inhibitor (CDKI) p21 and of the specific cardiac proteins MLC2 and cTnI. Analysis of the basal level of cAMP and cGMP, in partially differentiated cells treated with curcumin, showed an increase in the level ofcGMP and a decrease in those of cAMP. The rate of cAMP/cGMP degradation was increased after long term treatment with curcumin, while, direct administration of curcumin to cell extracts results in suppression of cAMP and cGMP, probably related to curcumin induced phosphodiesterase degradation [255]. Finally, an in vitro model of hypoxia and reoxygenation, which mimics ischemia/reperfusion injury, further confirms the protective effects of curcumin against cardiovascular injury [256]. Curcumin was revealed to significantly reduce cell loss, nuclei condensation and caspase 3 activity, induced by hypoxia and reoxygenation, protecting cells from mitochondrial dysfunction. In addition, curcumin inhibits Hypoxia-Inducible Factor-1α (HIF-1α) expression, directly activated by the cAMP-1 (Epac-1)/Akt pathway, increasing Epac1 levels, Akt phosphorylation and downregulating Erk1/2 and p38 phosphorylation. Overall, these effects strongly improve BMSC survival [256].

### 4.6. BPF

The potential role of bergamot polyphenols in modulating stem cell properties remains to be extensively evaluated. To date, the first interesting results were collected by analyzing the effects of the polyphenolic fraction of bergamot (BPF) on an experimental model of anthracycline-induced HF [223]. For the first time, BPF was observed to prevent eCSCs attrition, improving the number of resident c-kit^+^CD45^neg^CD31^neg^eCSCs and inhibiting 8-OHdG nuclear accumulation. Interestingly, these effects were associated with CM replenishment, recognized through an increasing number of newly formed BrdU^pos^ small myocytes. These data suggest that the direct ROS-scavenging properties of BPF, already discussed in the previous paragraph, lead to additional beneficial effects on the eCSC compartment by promoting the proliferation and differentiation of the eCSCs. Indeed, the maintenance of low ROS levels could guarantee the constitutive activity of the endogenous antioxidant enzymes thus ensuring the right redox status of the eCSC niches. In support of these findings, recent evidence shown that naringin, one of the most abundant flavonoids contained in BPF, protects human adipose-derived MSCs from the detrimental action of H_2_O_2_ which inhibits osteogenic differentiation. The protective effect of naringin is probably exerted through its action in modulating wnt signaling [257]. As extensively reported, wnt pathway is crucial for heart development and is probably inhibited in the adult heart [258], suggesting that the latter event may be beneficially modulated by BPF-derived naringin. Moreover, other hypothetical mechanisms leading to cardioprotective effects of citrus-derived flavonoids against HF were identified, such as an estrogenic like action which might induce progenitor cell mediated recovery [259]. Furthermore, in addition to the anti-oxidant actions, other mechanisms are currently being studied that participate in the mediation of the protective actions of BPF on the CSCs compartment, probably through paracrine effects and/or exosome involvement.

Interestingly, all these studies pointed out that the use of natural compounds could be involved in the cardiogenesis of different stem cell types by directly stimulating endogenous stem cells and their secretory activity and/or using conditioned media containing cytokines and signal modulators, increasing the level of some cardiac specific markers. The data suggest a possible future approach to obtain an optimal condition for efficient cardiac commitment with the use of natural inducers.

## 5. Conclusions

After many years, the belief that the heart was a terminally differentiated organ has been denied by groundbreaking discoveries about CM turnover and the existence of stem and progenitor cells located in the myocardium, which support its self-renewal potential. In this regard, stem cell therapy has gained particular attention, emerging as a novel approach to restore damaged myocardial tissue. Interesting pre-clinical studies reporting significant stem cell-mediated cardiac regeneration, encouraging subsequent first clinical trials. However, the beneficial effects of stem cell-based treatment on myocardial performance are still quite limited by the low engraftment and survival rate of the cells due to the microenvironment of the damaged site of the cardiac tissue. Therefore, boosting the efficacy of stem cell repair is essential, as well as better understanding the heart endogenous reparative mechanisms and their interactions with stem cell regenerative properties, making cardiac regenerative therapy an effective therapeutic tool. The indirect stimulation of cardiovascular commitment and paracrine regulation in various stem cell populations opens a new scenario for cardiac stem cell therapy.

Interestingly, pharmacological and non-pharmacological preconditioning represents a novel and efficient technique for stimulating the secretory activity of stem cells and drug-mediated activation or inhibition of pathways, which can modify stem cell physiology by improving cell survival, engraftment and regeneration.

Although limited data is available, the targeted use of certain natural compounds from plant extracts is emerging as a useful tool for orchestrating stem cell homing, engraftment and survival in heart diseases.

The upcoming challenges will concern a deeper understanding of the underlying mechanisms by which natural compounds can modulate stem cell cardiovascular lineage specification, and the main role played by stimulating their secretory activity and regulating the paracrine signaling. It will also be necessary to investigate whether the effects observed in experimental models of myocardial diseases are retained in humans with a sufficient degree of therapeutic efficacy without toxic effects.

CVDs represent complex disorders characterized by several disease patterns and pathologic mechanisms that make it difficult and ineffective to provide a uniform therapeutic intervention protocol. Therefore, future strategies should aim at identifying the optimal cell type, the specific stimulation/differentiation protocol, dosage and delivery approach that are effective and safe allowing for personalized therapy. In this perspective, the use of nutraceuticals can contribute significantly to the optimization of stem cell therapy in the clinical practice also thanks to their overall safety profile, feasibility and wide availability.

## Data Availability

Data available in a publicly accessible repository.
